# Hexavalent Chromium Inhibited Zebrafish Embryo Development by Altering Apoptosis- and Antioxidant-Related Genes

**DOI:** 10.3390/cimb45080436

**Published:** 2023-08-18

**Authors:** Khoa Dang Dang, Chi Nguyen Quynh Ho, Huy Duc Van, Son Thanh Dinh, Quynh Thi Truc Nguyen, Tram Thi Thuy Nguyen, Xuyen Thi Ngoc Kien, Tuyet Van Dao, Hung Viet Nong, Minh Thai Nguyen, Chung Chinh Doan, Son Nghia Hoang, Thao Thi Phuong Nguyen, Long Thanh Le

**Affiliations:** 1 Faculty of Biotechnology, Binh Duong University, Thu Dau Mot City 750000, Vietnam; dangdangkhoacm@gmail.com; 2 Biotechnology Department, Graduate University of Science and Technology, Vietnam Academy of Science and Technology, Hanoi 100000, Vietnam; quynhchihonguyen@gmail.com (C.N.Q.H.); nttquynh201096@gmail.com (Q.T.T.N.); ttramnt@gmail.com (T.T.T.N.); ntminh062@gmail.com (M.T.N.); doanchinhchung@gmail.com (C.C.D.); hoangsonitb@gmail.com (S.N.H.); 3 Institute of Tropical Biology, Vietnam Academy of Science and Technology, Ho Chi Minh 700000, Vietnam; sonthanhdinh@gmail.com; 4 Faculty of Biology and Biotechnology, University of Science, Ho Chi Minh 700000, Vietnam; huy.biotech@gmail.com; 5 Ho Chi Minh City University of Physical Education and Sports, Ho Chi Minh 700000, Vietnam; xuyenntk@upes.edu.vn; 6 Environmental Industry Institute, Ministry of Industry and Trade, Hanoi 100000, Vietnam; tuyetdv@gmail.com (T.V.D.); nongviethung@gmail.com (H.V.N.)

**Keywords:** apoptosis, chromium, embryo development, zebrafish

## Abstract

This study aimed to assess the effects of hexavalent chromium on zebrafish (*Danio rerio*) embryo development. The zebrafish embryos were treated with solutions containing chromium at different concentrations (0.1, 1, 3.125, 6.25, 12.5, 50, and 100 µg/mL). The development of zebrafish embryos was estimated by the determination of survival rate, heart rate, and the measurement of larvae body length. Real time RT-PCR and Western blot were performed to assess the expression of apoptosis- and antioxidant-related genes. The results showed that the reduced survival rate of zebrafish embryos and larvae was associated with an increase in chromium concentration. The exposure of higher concentrations resulted in a decrease in body length of zebrafish larvae. In addition, a marked increase in heart rate was observed in the zebrafish larvae under chromium treatment, especially at high concentrations. The real-time RT-PCR analysis showed that the transcript expressions for cell-cycle-related genes (*cdk4* and *cdk6*) and antioxidant-related genes (*sod1* and *sod2*) were downregulated in the zebrafish embryos treated with chromium. Western blot analysis revealed the upregulation of Caspase 3 and Bax, while a downregulation was observed in Bcl2. These results indicated that hexavalent chromium induced changes in zebrafish embryo development by altering apoptosis- and antioxidant-related genes.

## 1. Introduction

The element of chromium can be found in nature as a mineral. Depending on the chemical valence and dosage, it can act as a carcinogenic agent, as well as an important micronutrient [1]. The USA Environmental Protection Agency lists chromium as one of the eight most prevalent heavy metal contaminants because it is regarded as a harmful element [2]. Trivalent and hexavalent chromium are the two main valence states of chromium, and trivalent chromium is much less toxic than hexavalent chromium [3]. Aquatic animals exposed to hexavalent chromium show more immediate or chronic impacts, including dramatic death, growth inhibition, and decreased progeny production [4].

Hexavalent chromium is transported into the cells following the divalent anion concentration gradient through the intracellular chloridephosphate anionic channel [5]. Tetravalent chromium is produced when hexavalent chromium interacts with glutathione/glutathione synthetase in chloride channels in the cytoplasm and organelle membrane. They spread to the mitochondria and nucleus, where they can induce DNA disruption [5]. Hexavalent chromium prenatal exposure causes early reproductive senescence in the F1 rat offspring by enhancing the apoptosis of germ cells and increasing the disintegration of germ cell cysts [6]. Male mice somatic cells and spermatogonial stem cells underwent mitochondria-dependent apoptosis when exposed to hexavalent chromium. Additionally, the physiological processes of mouse TM3 Leydig cells and TM4 Sertoli cells were likewise hampered by hexavalent chromium, which also interfered with the differentiation and self-renewal mechanisms of spermatogonial stem cells [7]. Hexavalent chromium induced the alteration of rat oocyte development by increasing oxidative stress, breaking DNA double-strands, disrupting microtubules, and segregation of abnormal chromosomes [8]. Trivalent chromium causes the reduction in blastocyst ratio, a decrease in the cell numbers in an embryo, generation of abnormal lineage differentiation, and the enhancement of oxidative stress and apoptosis [9]. Hexavalent chromium accelerated apoptosis in trophoblasts, the vascular endothelium of the metrial glands and yolk sac epithelium through caspase-3 and p53-dependent pathways [10]. Hexavalent chromium decreases the expression of Bcl-2, Bcl-XL, and XIAP in the placenta while upregulating apoptosis in the labyrinth and basal zones [10].

Investigations on the toxicity of different heavy metals have also been conducted with a variety of animal models; however, the zebrafish has emerged as the main model for in vivo tests [11]. A chromium-contaminated adult zebrafish diet decreases the viability of zebrafish offspring [12]. Chromium induces changes in the metabolism of zebrafish larvae and causes neurotoxicity [13]; moreover, chromium treatment also alters the metabolome and microbiome of zebrafish, which is associated with neurotoxicity [14]. Zebrafish embryos exposed to chromium exhibit aberrant embryonic development and teratogenic consequences, including severe heart abnormalities [1]. During zebrafish development, chromium increases the developmental toxicity of graphene oxide [15]. However, the expression of apoptosis-related genes and antioxidant-associated genes has not been well characterized in zebrafish embryos subjected to chromium exposure. This study applied zebrafish as a model to estimate the effects of chromium on zebrafish embryo and larvae development. The objective of this study is to evaluate the alteration in the expression of apoptosis- and antioxidant-associated transcripts and proteins that are associated with the changes in zebrafish embryogenesis.

## 2. Materials and Methods

### 2.1. Breeding and Embryo Collection

E3 medium was applied to maintenance zebrafish embryos and larvae [16]. A 60 × stock solution was prepared by dissolving 34.8 g NaCl, 1.6 g KCl, 5.8g CaCl_2_.2H_2_O, and 9.78 g MgCl_2_.6H_2_O in 1.95 L H_2_O. The pH of the stock solution was adjusted to 7.2. The volume of the stock solution was adjusted to 2 L by adding H_2_O. A 1 × E3 medium was prepared by diluting 16.5 mL 60× stock in 1 L H_2_0. A total of 100 µL of 1% methylene blue was subsequently added to this medium. The adult zebrafish were kept throughout the experiments in an aquarium recirculation system under a 14/10 h light/dark cycle. The water temperature was maintained at 28 ± 0.5 °C. The males and females were chosen based on secondary sexual traits and placed in breeding tanks with mesh at the bottom on the night prior to spawning. The next day, 30 min after the light turned on, the zebrafish embryos were collected from the breeding tanks and were washed by E3 medium. The alive zebrafish embryos with normal morphology were used to perform the following experiments.

### 2.2. Chromium Exposure

The zebrafish embryos were collected and raised at 28 °C in a 1 × E3 medium over a 14:10 h light/dark cycle. One hour after fertilization, the zebrafish embryos were collected and divided into treatment groups and exposed to solutions containing chromium at different concentration (0.1, 1, 3.125, 6.25, 12.5, 50, and 100 µg/mL). A total of 50 zebrafish embryos were selected and transferred onto a 100 mm Petri dish. At 24 h intervals, the solutions were changed. To keep the surviving embryos from being contaminated, the dead embryos were taken out of the exposure chambers.

### 2.3. Effect of Chromium on Zebrafish Embryo Development

To estimate embryo development, the number of live embryos were determined from day 1 to day 7. The zebrafish embryos were checked under a stereomicroscope to discard the deformed embryos, the degenerated and dead embryos, and the embryos with morphological abnormalities. The survival rates of the treatment group and control group were recorded.

### 2.4. Heart Rate and Body Length Evaluation

The embryos on day 3 were brought to room temperature and left stable for 30 min in order to assess their heart rates. Ten embryos were randomly chosen for each group, and, under a stereomicroscope, their heart rates were recorded four times for a total of 15 s. Ten larvae were chosen at random on day 3 to determine the body length. Using a digital camera mounted on a microscope, the zebrafish larvae were captured with a ruler. Image J software 1.53c (National Institutes of Health, Bethesda, MD, USA) was applied to measure the length of the larvae along the body axis. To set the measurement scale, a line was drawn between two points of known distance of a ruler on the photograph, then by going to ***Analyze*** and choosing ***Set Scale***. Typing the known distance and units of measure in the appropriate boxes of the ***Set Scale*** window. To measure the zebrafish larvae body length, a line was drawn along the body axis, then by going to ***Analyze*** and choosing ***Measure to*** transfers the values to a data window. These data were used to estimate the zebrafish larvae body length.

### 2.5. Quantitative Real-Time RT-PCR

#### 2.5.1. Total RNA Extraction

The E.Z.N.A.^®^ Total RNA Kit I (R6834-02, Omega Bio-tek, Norcross, GA, USA) was used to extract total RNA. The embryos were homogenized with 350 µL TRK Lysis Buffer (Omega Bio-tek, Inc., Norcross, GA, USA) and centrifuged at maximum speed (≥12,000× *g*) for 5 min. The cleared supernatant was transferred to a clean 1.5 mL microcentrifuge tube. One volume of 70% ethanol was added to this tube and vortexed to mix thoroughly. A HiBind^®^ RNA Mini Column (Omega Bio-tek, Inc., Norcross, GA, USA) was inserted into a 2 mL collection tube. A 700 μL sample was transferred to the HiBind^®^ RNA Mini Column and centrifuged at 10,000× *g* for 1 min. The filtrate was discarded. A total of 500 μL of RNA wash buffer I was added to this tube and centrifuged at 10,000× *g* for 30 s. The filtrate was discarded. A total of 500 μL of RNA wash buffer II diluted with 100% ethanol was added to this tube and centrifuged at 10,000× *g* for 1 min. The filtrate was discarded. The empty HiBind^®^ RNA Mini Column was centrifuged at maximum speed for 2 min to dry the column. The HiBind^®^ RNA Mini Column was transferred to a clean 1.5 mL microcentrifuge tube. A total of 100 μL nuclease-free water was added to this tube and centrifuged at maximum speed for 2 min. The NanoVue Plus spectrophotometer (GE Healthcare, Arlington Heights, IL, USA) was used to evaluate the RNA sample’s amount and quality.

#### 2.5.2. Real Time RT-PCR Performance

The PCR reaction was conducted: 1 μL of total RNA, 2 μL of primers (including forward and reverse primers), 10 μL of Mix Ro-Lox, and 1 μL of RTAse (all in a total volume of 20 μL in each reaction). The PCR reactions were performed with 1 cycle of 45 °C for 15 min; 1 cycle of 95 °C for 2 min; 40 cycles of 95 °C for 10 s and 62 °C for 15 s; and 71 cycles of 60 °C for 30 s. The primers were as follows: bcl2-F: 5′-GGA TGA CTG ACT ACC TGA ACG G-3′ and bcl2-R: 5′-GTA TGA AAA CGG GTG GAA CAC A-3′; bax-F: TGC CTT TTA TTA GAA AGA CCT GCA T-3′ and bax-R: TCC AGC AAG GAA AAC TCC AAC T-3′; caspase 3-F: 5′-ATG AAC GGA GAC TGT GTG GA-3′ and caspase 3-R: 5′-GTA TCT GAA GGC ATG GGA TTG A-3′ [17], cdk4-F: 5′-GTA TGA GCC AGT AGC AGA GAT CG-3′ and cdk4-R: 5′-AGT TGT GGT GGG AAA GAG TGA C-3′; cdk6-F: 5′-GTA CAA GGC TCG GGA TTT G-3′ and cdk6-R: 5′-CTC TGG GGC TCG ATA CCA TA-3′; cdk21-F: 5′-CTG AAG CCT GAC AAT GTG CT-3′ and cdk21-R: 5′-GCA AGC CAA TTA CCT CAA AGA-3′ [18]; and elongation factor 1 alpha (EF1α): EF1α-F: 5′-GTA CTA CTC TTC TTG ATG CCC-3′ and EF1α-R: 5′-GTA CAG TTC CAA TAC CTC CA-3′ [19]. The 2^−ΔΔCt^ method was applied for Ct value analysis [20].

### 2.6. Western Blot

The embryos were collected and treated with Optiblot LDS Sample Buffer (ab119196, Abcam, Cambridge, MA, USA). Protein was added to each well of the Precast Gel SDS-PAGE 4–12% (ab139596, Abcam, Cambridge, MA, USA) at an equal proportion. The gel was run in Optiblot SDS Run Buffer (ab119197, Abcam, Cambridge, MA, USA) for 2 h at 50 V. The protein was transferred to a PVDF membrane (ab133411, Abcam, Cambridge, MA, USA), and the membrane was blocked overnight at 4 °C with a blocking buffer (ab126587, Abcam, Cambridge, MA, USA). The membrane was incubated with primary antibodies in a blocking buffer overnight at 4 °C. The anti-caspase 3 antibody (ab44976, Abcam, Cambridge, MA, USA), anti-Bax antibody (ab53154, Abcam, Cambridge, MA, USA), and anti-bcl-2 antibody (ab196495, Abcam, Cambridge, MA, USA) were employed at a 1:5000 dilution. The anti-GAPDH antibodies (ab181602, Abcam, Cambridge, MA, USA) were used at a 1:10,000 dilution. The membrane was washed three times with TBST for 10 min each. The membrane was incubated with a secondary antibody in a blocking buffer at room temperature for 1 h. Goat anti-mouse IgGs (HRP) (ab6789, Abcam, Cambridge, MA, USA) and goat anti-rabbit IgGs (HRP) (ab6721, Abcam, Cambridge, MA, USA) were used to detect the beta-actin antibody and other primary antibodies, respectively. The blots were visualized using the ECL Western Blotting Substrate Kit (ab65623, Abcam, Cambridge, MA, USA). Imaging was carried out with an X-ray film. The quantification of the exposed bands was performed by using Image J (National Institutes of Health, Bethesda, MD, USA).

### 2.7. Statistical Analysis

All data were expressed as the mean ± standard deviation. The data were analyzed for statistical significance by one-way ANOVA, where a *p* < 0.05 was considered statistically significant.

## 3. Results

### 3.1. Survival and Hatching Rate

**Figure 1:**
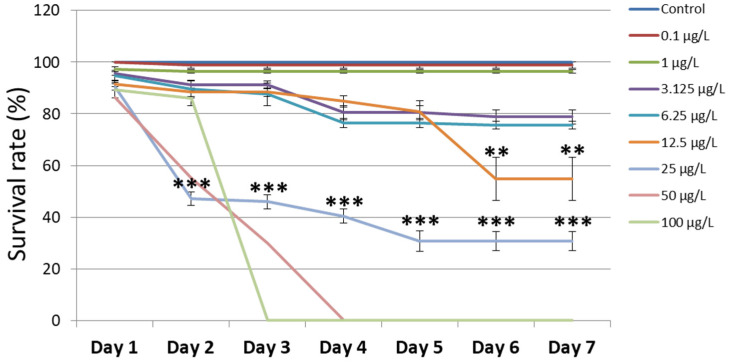
The survival rate of zebrafish embryos and larvae that underwent chromium treatment (*n* = 5). The embryos treated with a low concentration of chromium showed the same survival percentage from day 1 to day 7. The increase in chromium concentration causes the reduced development of zebrafish embryos. ** Indicates a significant difference from the group of 12.5 µg/L chromium treatment compared to the other groups (*p* < 0.01). *** Indicates a significant difference from the group of 25 µg/L chromium treatment compared to the other groups (*p* < 0.001).

The zebrafish embryo development is shown in Figure 1 and Supplementary Data S1. In order to identify the chromium concentration that might result in developmental abnormalities, zebrafish embryos were subjected to a wide range of chromium concentrations (0.1 µg/L to 100 µg/L). The embryos treated with 0.1 µg/L and 1 µg/L of chromium showed the same survival percentage from day 1 to day 7 (98.8% and 96.4%, respectively). The survival percentage decreased at day 2 in embryos treated with the higher concentration of chromium (3.125 µg/L, 6.25 µg/L, and 12.5 µg/L). The survival percentage of embryos treated with 25 µg/L of chromium decreased to 47.2% on day 2 and continued to reduce to 30.8% on day 7. Interestingly, the hatching of the embryos in this group was observed to be delayed until day 5. Treatment with 50 µg/L and 100 µg/L of chromium showed a strong decrease in zebrafish embryo development by demonstrating the developmental blocks at day 4 and day 3, respectively. Moreover, the lethal doses (LD50) at day 3 and day 7 were 32.3 µg/L and 16.4 µg/L, respectively. The control group showed normal development in accordance with Kimmel’s description [21]. These results demonstrated that an increase in chromium concentration results in a decrease in zebrafish embryo development.

### 3.2. Body Length

**Figure 2:**
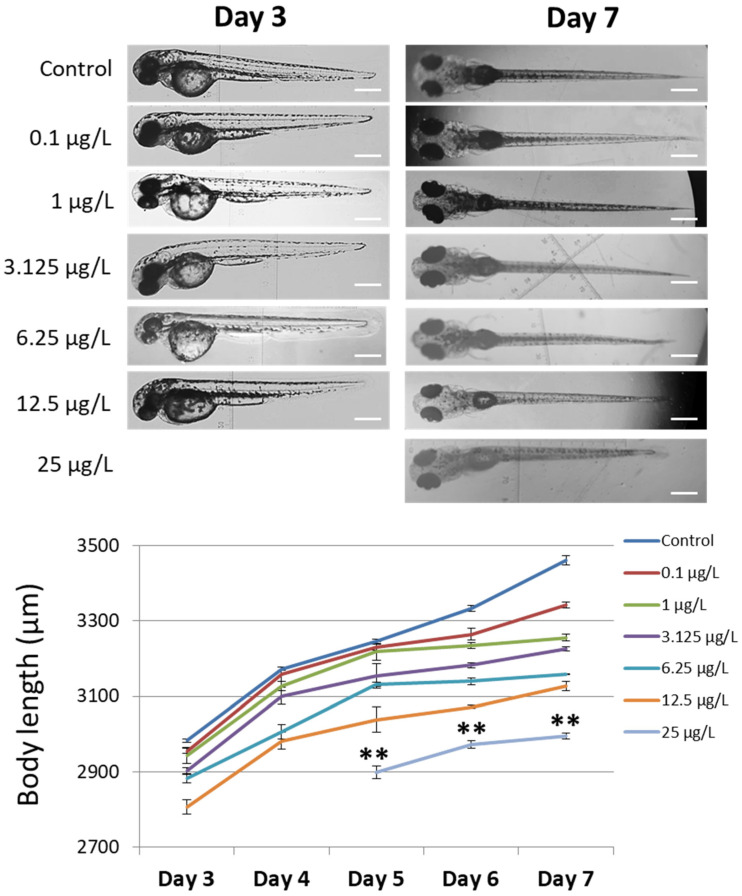
The body length of zebrafish larvae that underwent chromium treatment (*n* = 5). The measurement was performed in larvae from day 3 to day 7. The increase in chromium concentration was responsible for the inhibition of body length development of zebrafish larvae. Treatment with 25 µg/L of chromium results in hatching delay in zebrafish embryos. Scale bar = 200 µm. ** Indicates a significant difference from the group of 25 µg/L chromium treatment compared to the other groups (*p* < 0.01).

The degree of the larvae development was estimated by the measurement of body lengths (Figure 2 and Supplementary Data S2). The body length was assessed in larvae treated with a range of chromium concentrations (0.1 µg/L to 25 µg/L). As seen in Figure 2, a high concentration of chromium gives rise to a reduction in larvae body length. The body length of larvae in the control group increases from 2982 µm to 3461 µm in a time-dependent manner (day 3 to day 7). During development, the larvae in groups treated with chromium (0.1 µg/L to 12.5 µg/L) showed a lower body length than did those in the control group. The hatching delay of the embryos treated with 25 µg/L of chromium led to low growth of the larvae from day 5 to day 7 (2898 µm to 2996 µm, respectively). These results indicated that the increase in chromium significantly inhibited zebrafish larvae growth.

### 3.3. Heart Rate

**Figure 3:**
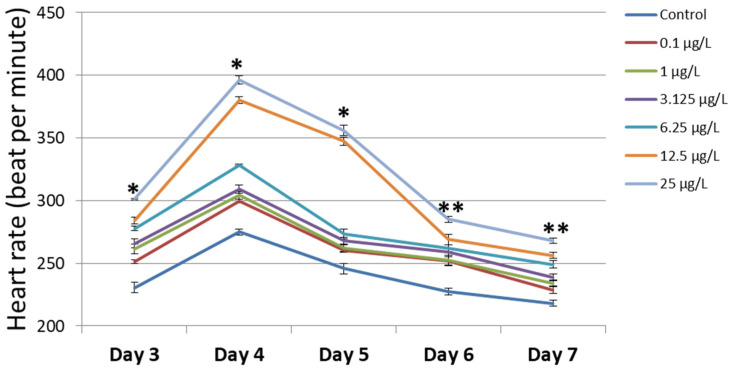
The heart rate of zebrafish larvae that underwent chromium treatment (*n* = 5). The measurements were performed in the larvae from day 3 to day 7. The increase in chromium concentration causes the increase in heart rate in zebrafish larvae. * Indicates a significant difference from the control group compared to the groups of 12.5 µg/L and 25 µg/L chromium treatment (*p* < 0.05). ** Indicates a significant difference from the control group compared to the other groups (*p* < 0.01).

The effect of chromium on cardiac function was evaluated by the recording of heart rates. The heart rate increased from day 3 to day 4 and gradually decreased from day 4 to day 7 in both the control and chromium-treated groups (Figure 3 and Supplementary Data S3). The larvae treated with chromium showed higher heart rates than the control group. These results showed that chromium treatment induced an increase in heart rate in zebrafish larvae.

### 3.4. Assessing the Transcript Expression of the Cell-Cycle-Related Genes, Oxidative-Stress-Related Genes, and Apoptosis-Related Genes

**Figure 4:**
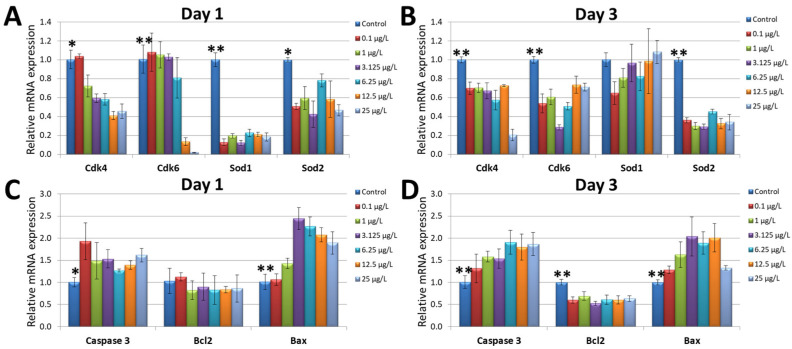
The transcript expression of cell-cycle-related genes (*cdk4*, *cdk6*), antioxidant-related genes (*sod1*, *sod2*), and apoptosis-related genes (*caspase 3*, *bcl2*, and *bax*). The experiments were repeated at least 3 times. (**A**,**C**) The real-time RT-PCRs performed on the zebrafish embryos on day 1. (**B**,**D**) The real-time RT-PCRs performed on the zebrafish embryos on day 3. ** Indicates a significant difference from the control group compared to the other groups (*p* < 0.01); * indicates a significant difference from the control group compared to the other groups (*p* < 0.05).

To examine the potential causes of harmful effects by chromium, real-time RT-PCR was conducted to assess the transcript expression of the cell-cycle-related genes, oxidative-stress-related genes, and apoptosis-related genes in the zebrafish embryos on day 1 and day 3. The results demonstrated that, after treatment with chromium concentrations at or above 1 µg/L (Figure 4A), the expression of *cdk4* transcripts was reduced when compared to the control group. However, all chromium-treated groups showed lower *cdk4* expressions than the control group at day 3. The expression of *cdk6* was markedly decreased in the embryos treated with 12.5 µg/L of chromium and 25 µg/L of chromium on day 1. On day 3, the recovery of *cdk6* expression was found in these groups. The chromium-treated groups showed a lower expression of cdk4 compared to the control group on day 3. The expression of *sod1* was increased in the chromium-treated groups on day 3 after downregulating on day 1. The *sod2* transcript expression in chromium-treated groups was lower than in the control group on day 1 and day 3.

The real-time RT-PCR also indicated that the transcript expressions of *caspase 3* and *bax* were upregulated in the embryos exposed to chromium on day 1 (Figure 4C). However, there was no significant difference of *bcl2* transcript expression between the embryos in the control group and chromium-treated groups. The increase in *caspase 3* and *bax* transcript expression was also observed in the embryos that had undergone 3 days of chromium treatment, and the down regulation of *bcl2* transcript was also determined in these embryos (Figure 4D).

### 3.5. Assessing the Apoptosis-Related Protein Expression

**Figure 5:**
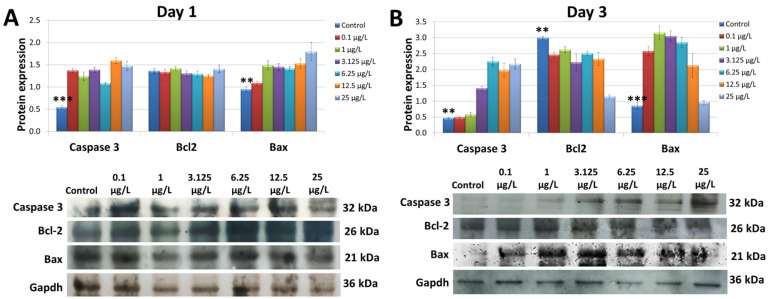
The expression of the apoptosis-related proteins (Caspase 3, Bcl2, and Bax) (*n* = 3). (**A**,**B**) The Western blot analysis that was performed on the zebrafish embryos on day 1 and day 3. *** Indicates a significant difference from the control group compared to the other groups (*p* < 0.001); ** indicates a significant difference from the control group compared to the other groups (*p* < 0.01).

Western blot analysis was applied to estimate the expression of the Caspase3, Bcl2, and Bax proteins. As seen in Figure 5A and Supplementary Data S4, the chromium exposure for day 1 induced an increase in the expression of Caspase 3 and Bax proteins when compared to the control group. The control group and chromium-treated group showed a similar Bcl2 expression in the embryos. The Caspase 3 upregulation was found in embryos treated with higher concentrations of chromium (3.125 µg/L, 6.25 µg/L, and 12.5 µg/L). Bax protein expression was also increased in the embryos that had undergone chromium exposure for 3 days. Nonetheless, the Bcl2 protein expression was reduced in the embryos that had undergone 3 days of chromium treatment (Figure 5B and Supplementary Data S4).

## 4. Discussion

In this study, we evaluated the effect of chromium on the development of zebrafish embryos and larvae. According to the findings, for the chromium treatment at low doses (0.1 g/L and 1 g/L), there were no significant effects on embryo survival throughout the process of embryonic development. However, the chromium exposure at higher concentrations (≥3.125 µg/L) decreased the survival of the zebrafish embryos from day 1 to day 7. These findings contribute to our understanding of how chromium affects the development of zebrafish embryos and larvae. It was found that 25 µg/L of chromium is the highest concentration at which zebrafish embryos can develop (up to day 7). With the lower concentrations of chromium exposure, embryo hatching took place on day 3, while the hatching of embryos under a 25 µg/L chromium treatment occurred on day 5. Hatching is the result of a combination of many factors, including osmosis, the presence of secreted hatching enzymes, and the lashing movement of the embryonic tail [22]. The changes in embryo size resulted in abnormalities in the hatchings [23]. In the present work, an increase in chromium concentration was associated with a decrease in the body length of zebrafish larvae from day 3 to day 7; this was especially the case in the group treated with 25 µg/L of chromium. This result revealed that a high concentration of chromium induces a delay in zebrafish embryo hatching.

Heart rate is an important parameter in studies of the physiology and developmental biology of fish [24]. This is because they are particularly sensitive to environmental changes, especially exposures to heavy metals [25]. Metal intoxication causes abnormalities in the cardiovascular systems of fish embryos, including changes in heart rate and cardiac activity, an increase in mortality, and deformations in the vertebral column [23,25]. In the present investigation, the heart rate of the zebrafish embryos correlated with an increase in chromium concentration. This change is due to the response of fish when they are under heavy metal exposure [25,26,27]. This result also suggested that chromium treatment induces an increase in the heart rate of zebrafish embryos.

SODs are crucial enzymes found in all living things that use oxygen. They catalyze the transformation of superoxide into oxygen and hydrogen peroxide [28]. It is essential for preventing the creation of ROS by healthy cells during heavy metal exposure [29]. Long-term heavy metal exposure can cause oxidative stress, which increases the generation of ROS and induces the apoptosis of zebrafish larvae [30]. The current study showed that, on day 1, the mRNA level of *sod1* and *sod2* was reduced in the zebrafish embryos under chromium treatment. However, only *sod2* transcript expression was maintained at a low level, while the recovery of *sod1* mRNA was observed on day 3. These results indicate that chromium exposure alters the *sod1* and *sod2* transcript expressions, which disrupts the antioxidant defense system in zebrafish embryos. The members of the cdk family play crucial roles in regulating cell division and modulating transcription, which alter the cell proliferation and process of apoptosis [17,18]. Cdk4 and Cdk6 control cell division by associating with Cyclin D. The changes in Cdk4 and Cdk6 can cause the dysregulation of cell division [19]. A decrease in the expression of *cdk4* and *cdk6* can result in apoptosis being induced [18]. Our study showed that chromium treatment attenuated *cdk4* and *cdk6* transcript expressions, thereby leading to the delay of zebrafish embryo development.

The investigation of apoptosis-related transcript levels revealed the triggering of apoptosis in zebrafish embryos. Previous studies have reported that the heavy metals that induce ROS generation can alter apoptosis-related gene expressions [20,30]. Real-time RT-PCR and Western blot analyses demonstrated that chromium exposure induced an upregulation of Caspase 3 and Bax, and the downregulation of Bcl2. Caspases are considered to be indicators of oxidative-stress-induced apoptosis in zebrafish embryos since they are crucial components of the apoptosis process. Caspase 3 is one of the convergence points of many different pathways that regulate apoptosis [31]. The Bcl2 family members play a crucial role in mediating the delicate balance between apoptosis and survival in eucaryotic cells [32]. The dysregulation of this signaling pathway can cause a variety of diseases related to development and degenerative disorders [33]. The present investigation showed that chromium treatment induced an increase in Caspase 3 expression. In addition, chromium-exposed embryos showed Bax upregulation and Bcl2 downregulation. These outcomes indicated that an increase in apoptosis occurred in embryos under chromium exposure, which itself gave rise to a decrease in their development.

The investigation of apoptosis-related transcript levels revealed the triggering of apoptosis in zebrafish embryos. Previous studies have reported that the heavy metals that induce ROS generation can alter apoptosis-related gene expressions [20,34]. Real-time RT-PCR and Western blot analyses demonstrated that chromium exposure induced an upregulation of Caspase 3 and Bax, and the downregulation of Bcl2. Caspases are considered to be indicators for oxidative-stress-induced apoptosis in zebrafish embryos since they are crucial components of the apoptosis process. Caspase 3 is one of the convergence points of many different pathways that regulate apoptosis [35]. The Bcl2 family members play a crucial role in mediating the delicate balance between apoptosis and survival in eucaryotic cells [36]. The dysregulation of this signaling pathway can cause a variety of diseases related to development and degenerative disorders [37]. The present investigation showed that chromium treatment induced an increase in Caspase 3 expression. In addition, chromium-exposed embryos showed Bax upregulation and Bcl2 downregulation. These outcomes indicated that an increase in apoptosis occurred in embryos under chromium exposure, which itself gave rise to a decrease in their development.

## 5. Conclusions

This study elucidated the effects of chromium on the development of zebrafish embryos. Chromium exposure resulted in abnormalities in the zebrafish embryo development such as reduced embryonic proportions, reduced body size, and induction of changes that altered heart rate. These alterations are associated with the changes in the expression of apoptosis- and antioxidant-related genes.

## Data Availability

All data sets obtained and analyzed during the current study are available in the manuscript.

## Supplementary Materials

The following supporting information can be downloaded at: https://www.mdpi.com/article/10.3390/cimb45080436/s1. Supplementary Data S1. Survival rate of zebrafish embryo; Supplementary Data S2. The body length of zebrafish larva; Supplementary Data S3. Heart rate; Supplementary Data S4. Western blot.

## Abbreviations

BCL2	B-cell leukemia/lymphoma 2 protein.
CDKs	Cyclin-dependent kinases
Ct value	Cycle threshold value
ECL	Enhanced chemiluminescence
IgG	Immunoglobulin G
LDS	Lithium dodecyl sulfate
PCR	Polymerase-Chain-Reaction
PVDF	Polyvinylidene fluoride
ROS	Reactive oxygen species
RT-PCR	Reverse transcription PCR
RTAse	Reverse transcriptase
SDS-PAGE	Sodium dodecyl sulfate polyacrylamide gel electrophoresis
SODs	Superoxide dismutase genes
