# Supplementary material for: Hexavalent Chromium Inhibited Zebrafish Embryo Development by Altering Apoptosis- and Antioxidant-Related Genes
Source: Curr Issues Mol Biol. 2023 Aug 18;45(8):6916–26. doi: 10.3390/cimb45080436 (PMC10453199; doi:10.3390/cimb45080436)

Supplementary data

1. Survival rate of zebrafish embryo

The number of embryo at day 1

No.	Chromium concentration (µg/L)								
	Control	0.1	1	3.125	6.25	12.5	25	50	100
1	100	100	100	94	94	92	92	88	84
2	100	100	98	96	88	92	92	90	88
3	100	100	96	94	96	92	92	80	94
4	100	100	94	96	98	88	90	80	82
5	100	100	98	98	98	94	86	94	98

The number of embryo in day 2

No.	Chromium concentration (µg/L)								
	Control	0.1	1	3.125	6.25	12.5	25	50	100
1	100	94.0	98	88	90	88	42	68	84
2	100	100	98	90	76	90	52	84	88
3	100	100	96	90	94	86	54	22	84
4	100	100	94	92	96	84	42	36	78
5	100	100	96	96	92	94	46	66	96

The number of embryo at day 3

No.	Chromium concentration (µg/L)								
	Control	0.1	1	3.125	6.25	12.5	25	50	100
1	100	94	98	88	84	88	40	58	0
2	100	100	98	90	72	90	52	60	0
3	100	100	96	90	94	86	52	12	0
4	100	100	94	92	96	84	40	16	0
5	100	100	96	96	92	94	46	4	0

The number of embryo at day 4

No.	Chromium concentration (µg/L)								
	Control	0.1	1	3.125	6.25	12.5	25	50	100
1	100	94	98	88	80	86	36	0	0
2	100	100	98	84	72	88	46	0	0
3	100	100	96	80	80	82	48	0	0
4	100	100	94	72	72	78	34	0	0
5	100	100	96	78	78	90	38	0	0

The number of embryo at day 5

No.	Chromium concentration (µg/L)								
	Control	0.1	1	3.125	6.25	12.5	25	50	100
1	100	94	98	88	80	86	28	0	0
2	100	100	98	84	72	84	38	0	0

3	100	100	96	80	80	78	40	0	0
4	100	100	94	72	72	66	30	0	0
5	100	100	96	78	78	90	18	0	0

The number of embryo at day 6

No.	Chromium concentration (µg/L)								
	Control	0.1	1	3.125	6.25	12.5	25	50	100
1	100	94	98	88	78	76	28	0	0
2	100	100	98	76	72	72	38	0	0
3	100	100	96	80	80	48	38	0	0
4	100	100	94	72	72	32	30	0	0
5	100	100	96	78	76	46	18	0	0

The number of embryo at day 7

No.	Chromium concentration (µg/L)								
	Control	0.1	1	3.125	6.25	12.5	25	50	100
1	100	94	98	88	78	76	28	0	0
2	100	100	98	76	72	72	38	0	0
3	100	100	96	80	80	48	38	0	0
4	100	100	94	72	72	32	30	0	0
5	100	100	96	78	76	46	18	0	0

2. The body length of zebrafish larva

The body length of zebrafish larva (µm) at Day 3

No.	Chromium concentration (µg/L)						
	Control	0.1	1	3.125	6.25	12.5	25
1	2974	2933	3039	2921	2918	2731	not hatching
2	2969	2922	2842	2927	2915	2737	not hatching
3	2987	2988	2994	2857	2842	2844	not hatching
4	2978	2973	2874	2872	2915	2914	not hatching
5	3005	2956	2969	2932	2821	2803	not hatching

The body length of zebrafish larva (µm) at Day 4

No.	Chromium concentration (µg/L)						
	Control	0.1	1	3.125	6.25	12.5	25
1	3185	3134	3092	3215	2964	2955	not hatching
2	3176	3081	3172	3161	3122	2847	not hatching
3	3188	3275	3147	2998	3000	3009	not hatching
4	3164	3163	3056	3064	2920	3013	not hatching
5	3146	3139	3167	3066	3022	3083	not hatching

The body length of zebrafish larva (µm) at Day 5

No.	Chromium concentration (µg/L)						
	Control	0.1	1	3.125	6.25	12.5	25
1	3283	3229	3111	3077	3135	2906	2987
2	3246	3200	3323	3007	3105	3013	2923
3	3223	3196	3231	3311	3122	3207	2906
4	3253	3273	3301	3222	3158	3139	2859
5	3218	3257	3129	3247	3135	2925	2816

The body length of zebrafish larva (µm) at Day 6

No.	Chromium concentration (µg/L)						
	Control	0.1	1	3.125	6.25	12.5	25
1	3371	3270	3212	3191	3156	3081	2964
2	3336	3339	3246	3203	3157	3092	2920
3	3328	3188	3239	3203	3152	3056	2955
4	3340	3302	3203	3179	3158	3064	3009
5	3292	3223	3275	3138	3077	3066	3013

The body length of zebrafish larva (µm) at Day 7

No.	Chromium concentration (µg/L)						
	Control	0.1	1	3.125	6.25	12.5	25
1	3490	3336	3294	3215	3167	3077	3049
2	3500	3328	3264	3269	3156	3148	2973

3	3470	3340	3239	3207	3157	3077	2989
4	3475	3393	3269	3224	3152	3148	2973
5	3371	3314	3212	3214	3158	3184	2994

3. Heart rate

The heart rate (beats per minute) at Day 3

No.	Chromium concentration (µg/L)						
	Control	0.1	1	3.125	6.25	12.5	25
1	233	255	266	270	278	280	300
2	226	253	263	266	284	290	303
3	220	250	270	275	275	290	302
4	230	246	246	256	276	280	300
5	244	252	262	262	275	280	300

The heart rate (beats per minute) at Day 4

No.	Chromium concentration (µg/L)						
	Control	0.1	1	3.125	6.25	12.5	25
1	280	297	297	300	330	370	389
2	278	300	298	305	330	380	390
3	268	302	306	310	328	384	405
4	272	304	314	320	326	382	403
5	276	295	305	310	328	385	395

The heart rate (beats per minute) at Day 5

No.	Chromium concentration (µg/L)						
	Control	0.1	1	3.125	6.25	12.5	25
1	255	260	270	276	278	360	370
2	240	262	266	270	286	340	360
3	244	266	259	265	266	345	350
4	256	256	256	260	265	344	348
5	233	258	258	268	271	348	350

The heart rate (beats per minute) at Day 6

No.	Chromium concentration (µg/L)						
	Control	0.1	1	3.125	6.25	12.5	25
1	226	242	264	270	270	279	290
2	220	256	258	260	267	270	280
3	237	263	248	255	257	260	280
4	230	250	244	254	257	260	285
5	224	247	247	257	260	275	290

The heart rate (beats per minute) at Day 7

No.	Chromium concentration (µg/L)						
	Control	0.1	1	3.125	6.25	12.5	25
1	211	232	230	240	250	260	270
2	216	222	234	244	255	260	270

3	219	238	244	245	255	260	270
4	224	228	228	230	240	250	270
5	220	223	233	235	245	250	260

4. Western blot

4. 1. The protein exposure at day 1

4.1.1. Caspase3

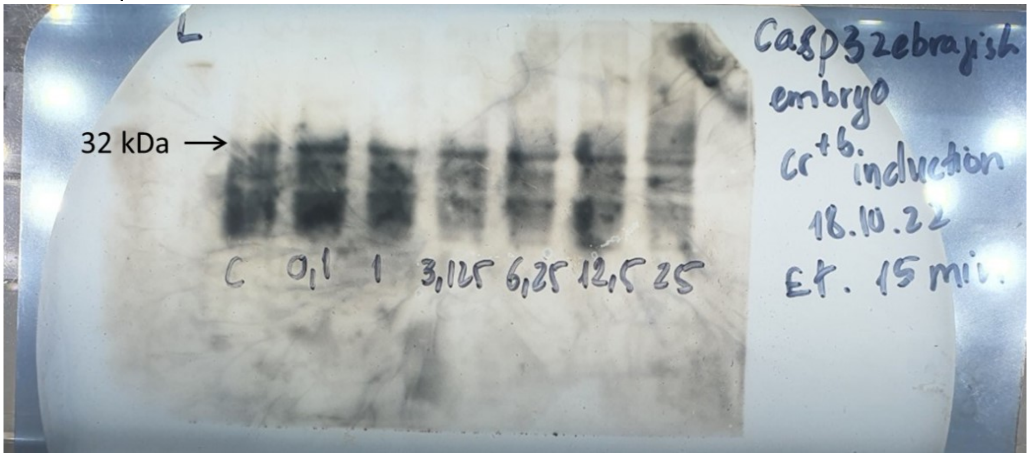

4.1.2. Bcl2

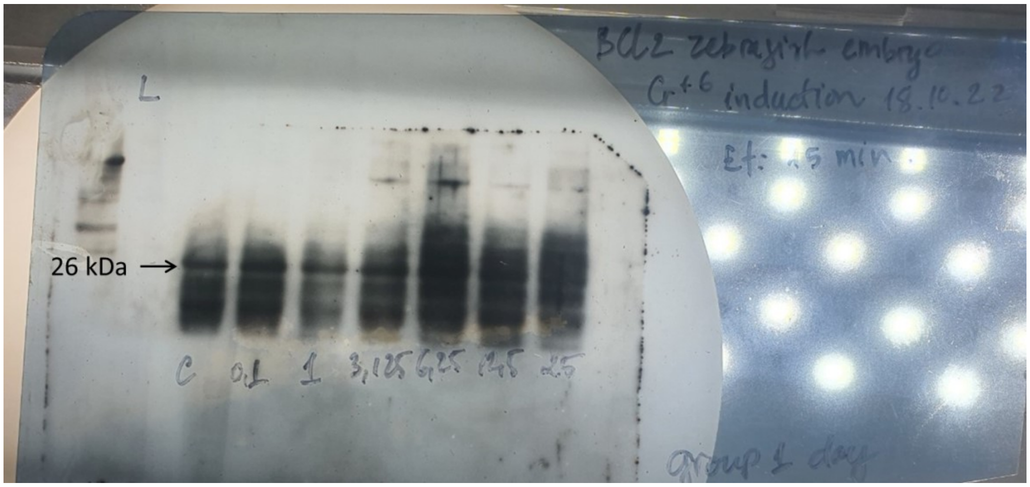

4.1.3. Bax

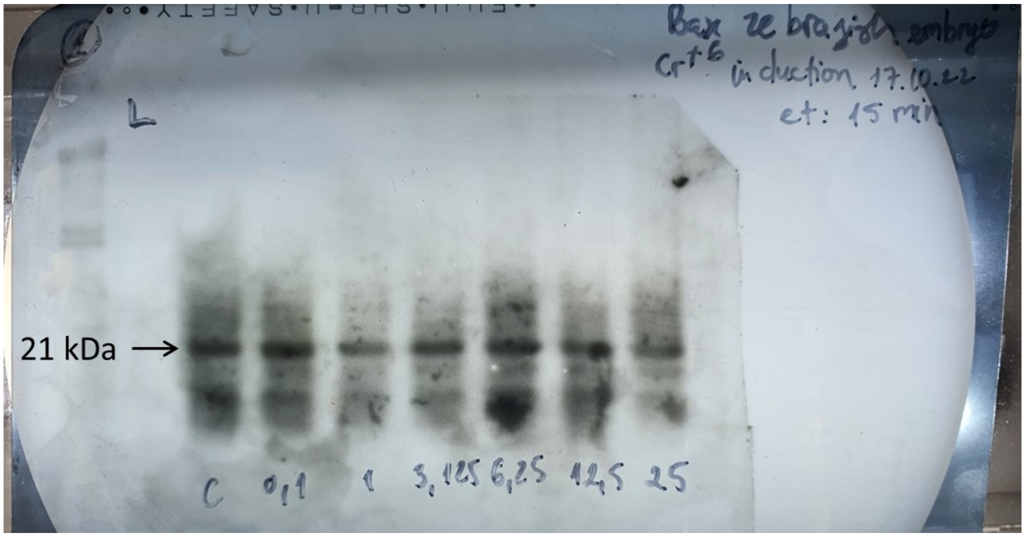

4.1.4. Gapdh

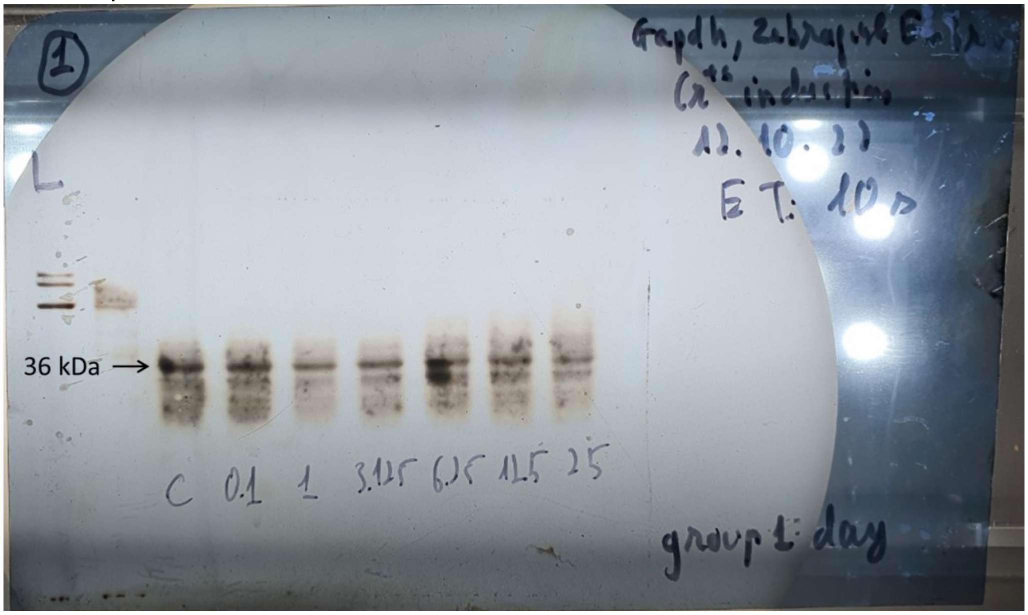

4.2. The protein exposure at day 3

4.2.1. Caspase3

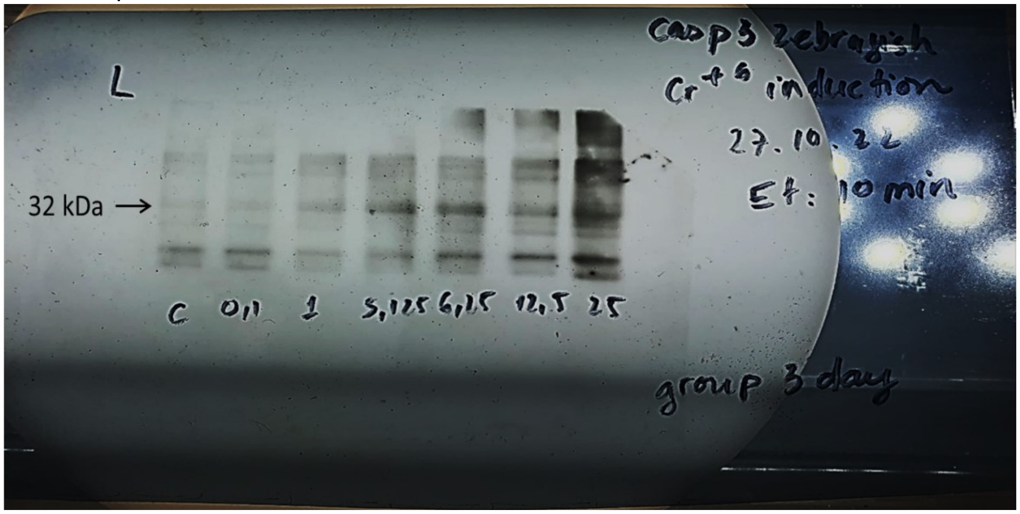

4.2.2. Bcl2

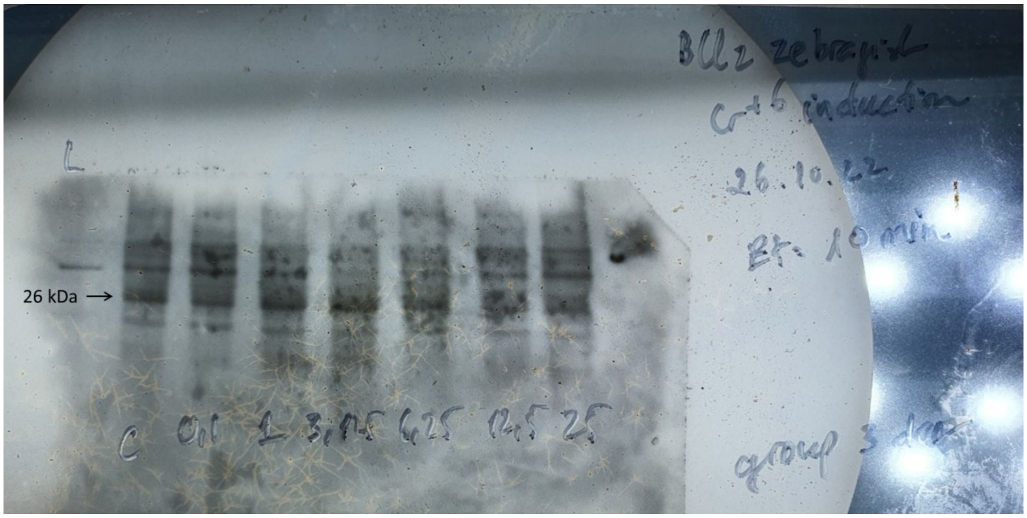

4.2.3. Bax

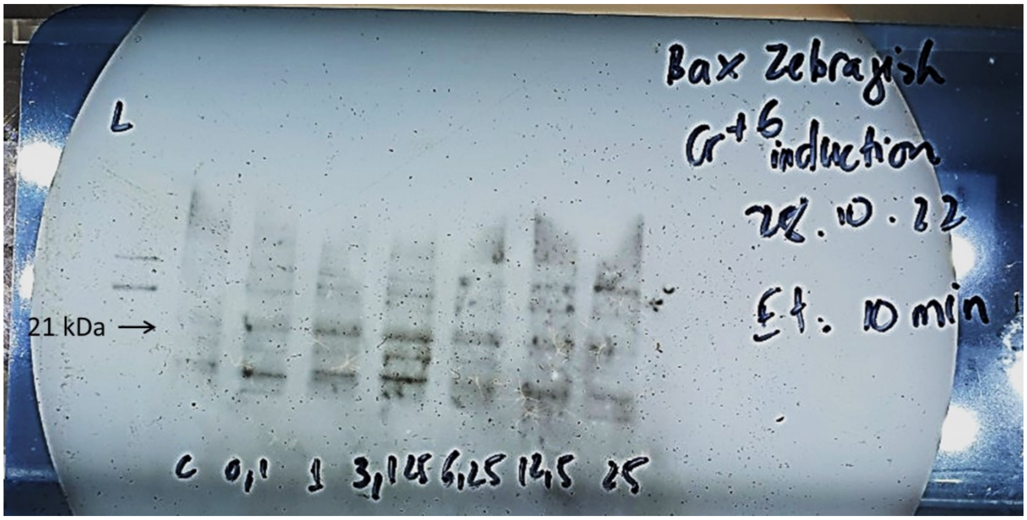

4.2.4. Gapdh

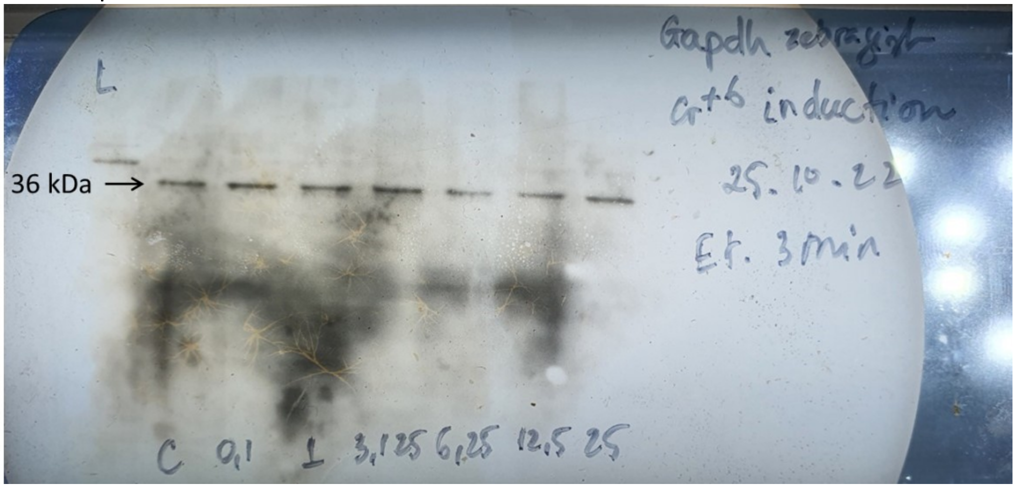

Supplement: Supplementary file 1 [file cimb-45-00436-s001.zip › cimb-2475955-supplementary.pdf]
